# UV-Curable Aliphatic Silicone Acrylate Organic–Inorganic Hybrid Coatings with Antibacterial Activity

**DOI:** 10.3390/molecules22060964

**Published:** 2017-06-09

**Authors:** Virginija Jankauskaitė, Algirdas Lazauskas, Egidijus Griškonis, Aistė Lisauskaitė, Kristina Žukienė

**Affiliations:** 1 Faculty of Mechanical Engineering and Design, Kaunas University of Technology, Studentų St. 56, LT-51424 Kaunas, Lithuania; lisauskait@yahoo.com (A.L.); kristina.zukiene@ktu.lt (K.Ž.); 2 Institute of Materials Science, Kaunas University of Technology, Baršausko St. 59, LT-51423 Kaunas, Lithuania; algirdas.lazauskas@ktu.lt; 3 Faculty of Chemical Technology, Kaunas University of Technology, Radvilėnų St. 19, LT-50254 Kaunas, Lithuania; egidijus.griskonis@ktu.lt

**Keywords:** silicone acrylate, silver nanoparticles, UV-curable, antibacterial

## Abstract

The most effective means to protect against bacterial invasion and to reduce the risk of healthcare-associated infections are antibacterial components synthesis. In this study, a novel process for the synthesis of organic–inorganic hybrid coatings containing silver nanoparticles is presented. Silver nanoparticles and polymer formation proceeds simultaneously through the in situ photoreduction of silver salt to silver nanoparticles and UV-crosslinking of bifunctional aliphatic silicone acrylate. The nanocomposite films with 0.5–1.43 wt % of silver nanoparticles concentration were obtained and investigated. The formation of silver nanoparticles in polymer matrix was confirmed via UV-visible spectroscopy, X-ray diffraction (XRD), Fourier transform infrared spectroscopy, scanning electron spectroscopy, and energy dispersive spectroscopy. Our investigations clearly show the formation of silver nanoparticles in silicone acrylate network. Direct photoreduction of silver salt by UV-radiation in the organic media produced silver nanoparticles exhibiting cubic crystal structure. The size of nanoparticles was determined to be near 20 ± 5 nm. The antibacterial activities of coatings were determined using the disc diffusion and direct contact methods. UV-curable silicone acrylate hybrid coatings exhibited antibacterial activity against harmful bacteria strains.

## 1. Introduction

Hybrid materials containing organic–inorganic constituents have gained increased interest due to their exceptional multifunctional properties. Different combinations allow the achievement of exceptional electrical, catalytic, antibacterial, radiation-resistant, optical, thermal, and mechanical properties. The potential applications of these materials can cover multiple fields, ranging from engineering to medical [1,2,3]. Hybrid organic–inorganic materials can be defined as nanocomposites obtained by intimate mixing of organic and inorganic components. The common route for the synthesis of these nanocomposites corresponds to the use of bridged and polyfunctional precursors, very convenient sol–gel chemistry, and hydrothermal synthesis [4]. More sophisticated techniques include the use of self-assembly, integrative synthesis routes, or templated growth by organic surfactants [5].

Organic–inorganic polymer hybrids are based on the combinations of polymer matrixes with metals, ceramics, or both, and can be prepared by a variety of methods for different applications [6,7]. The most widely used organic matrices for the incorporation of inorganic nanoparticles include poly(methyl methacrylate), poly(vinyl chloride), polycarbonate, poly(ethylene terephthalate), and polystyrene [8,9,10,11,12]. 

Silicone-modified acrylates form a new class of hybrid surface-modifying materials. These substances are not merely blends of silicone and acrylates. Instead, they are hybrids obtained by the chemical incorporation of silicone chains into basic acrylate structure [13]. Silicone-acrylates are obtained via the radical polymerization of acrylates or methacrylates with silicone macromers (i.e., long chains of polydimethylsiloxane) [14]. A single terminal reactive group (e.g., acrylic, methacrylic, or vinyl functional groups) is at the end of the chain. Hereby, in such a composition, silicones of low surface tension are combined with polyacrylates having relatively high surface tension. The strong surface orientation of the silicone chains creates a very strong repulsion effect against water and contaminants, such as oil and dirt, and bacterial adhesion.

Biofilm-producing pathogens are frequently associated with healthcare-associated infections that often need surgical intervention and the widespread use of broad-spectrum antibiotics [15]. The risk of such infections can be reduced by the modification of medical device materials with additives possessing antimicrobial activity with the ability to decrease bacterial adhesion.

Antibacterial components synthesis is the most effective means of protecting against colonization by bacterial pathogens and dangerous infections. Silver nanoparticles are a well-known potent antimicrobial agent. A number of different synthesis methods of silver nanoparticles have been developed, including chemical, photochemical, and thermal [16,17,18,19]. Specifically, the photo-induced synthesis strategy is very attractive, as it is a versatile and convenient process with distinguishing advantages, such as space-selective fabrication [20]. Direct photoreduction is a very important technique for nanoparticle synthesis, where the direct excitation of a metal source is employed for the formation of metal nanoparticles. Additionally, there is no need for reducing agents. Moreover, it can be employed in various media, including polymer coatings and thin films. Silver nanoparticles with their attractive physicochemical properties, size, and shape variety push the intensive research with great scientific interest into a wide range of applications, including biological, chemical, and physical sectors [21]. They are known to exert toxicity through mechanisms different from conventional antibiotics [22,23]; these include disruption of cell morphology, inactivation of vital cellular enzymes and proteins, DNA condensation [24], inhibition of ribosome interaction, accumulation at lethal concentration in cells [25], protein denaturation [26], modulation of cellular signaling, generation of reactive oxygen species, oxidative stress [27], loss of DNA replication, and depletion of adenosine triphosphate [28]. This can be especially useful to ensure a sterile environment in medical applications (e.g., surgical settings) by preventing bacteria from growing on or adhering to the surface [29,30].

In the present study, a simple and fast hybrid organic–inorganic polymer synthesis method is investigated. UV-cured aliphatic silicone acrylate coatings containing silver nanoparticles were formed via photo-polymerization technique. These coatings were characterized employing multiple analytical techniques.

## 2. Results and Discussion

### 2.1. Structure Analysis

**Figure 1:**
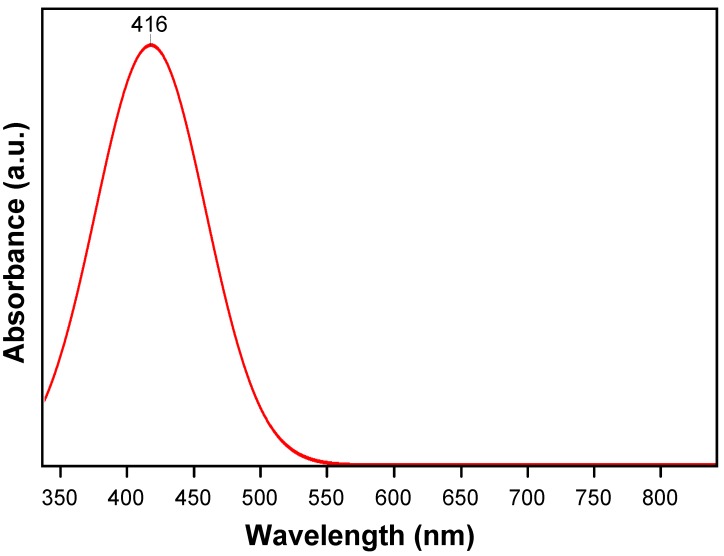
UV-visible light absorbance spectra of silver nanoparticles containing UV-cured silicone acrylate composite coating.

In order to confirm the presence of silver nanoparticles in the fabricated UV-cured silicone acrylate composite structure, UV-visible spectroscopy was used in the first instance. Figure 1 shows the UV-visible light spectrum of silicone acrylate-silver nanocomposite, presented as a Gaussian function which has a characteristic absorption peak at around 416 nm due to the surface-plasmon resonance band of the silver nanoparticles [31,32]. In most cases, the absorption peak is observed in the range of 410–460 nm due to surface plasmon resonance in silver nanoparticles. It is known that the absorption bandwidth of the plasmon band depends heavily on the size and interaction with the surrounding medium [33]. The band centered at 416 nm in the UV-visible light absorbance spectrum (337–840 nm) indicates the presence of silver nanoparticles in the polymer matrix, yielding a unique spectral fingerprint for plasmonic nanoparticles with a size corresponding to 20 ± 5 nm [34].

**Figure 2:**
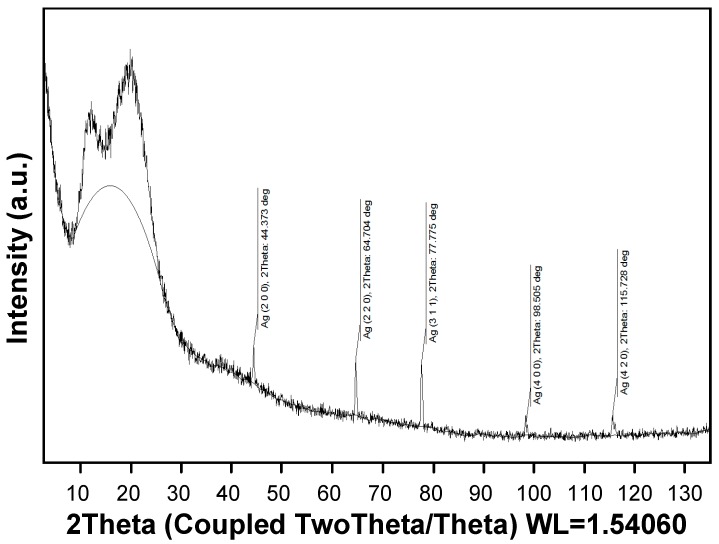
XRD pattern of silver nanoparticles containing UV-cured silicone acrylate composite coating with crystallographic plane orientations of silver phase indicated.

The structural peculiarities of synthesized silver nanoparticles were determined using X-ray diffraction (XRD). Figure 2 shows an XRD pattern of a UV-cured aliphatic silicone acrylate nanocomposite coating which contains silver nanoparticles. After the reduction process, silver nanoparticles crystallized into cubic crystal structure (as per JCPDS file no. 04-016-5006) with space group Fm-3m (group number = 225), and the (2 0 0), (2 2 0), (3 1 1), (4 0 0), and (4 2 0) crystallographic plane orientations were observable. The diffraction pattern also reveals two broad components in the range of 2θ = 8.0–28.0°, which are attributed to the amorphous structure of aliphatic silicone acrylate.

**Figure 3:**
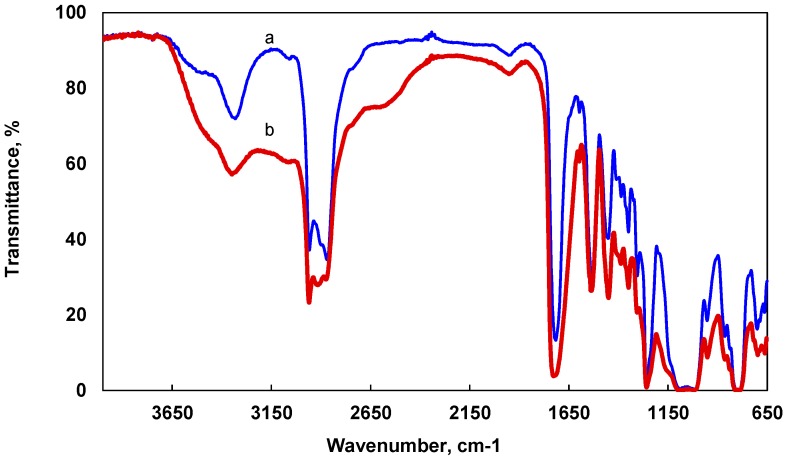
Fourier transform infrared (FTIR) spectra of UV-curable aliphatic silicone acrylate coating (**a**) without and (**b**) with 1.0 wt % of silver nanoparticles.

The chemical structure of UV-cured silicone acrylate nanocomposite was identified via FTIR spectroscopy (Figure 3). The sharp peak at 1720 cm^−1^ is attributed to the stretching vibration of C=O groups, and a peak at 1258 cm^−1^ is related to C–O stretching vibration of the acrylic moiety [35]. An absorption peak at 2960 cm^−1^ corresponds to C–H symmetric and asymmetric stretching of methyl and methylene groups. The presence of this group is also confirmed by peaks at 1450 cm^−1^ and 1387 cm^−1^, which are associated with a bending vibration of C–H groups.

The silicone part could be characterized by Si–CH_3_ symmetric deformation and stretching vibration at 1260 and 788 cm^−1^, respectively. The bands in the 1070–1015 cm^−1^ region are attributed to Si–O–Si stretching vibration [36,37,38,39]. There are no noticeable shifts in the peak positions when the silicone acrylate polymer without nanoparticles and polymer with embedded silver nanoparticles are compared, except for a broader and stronger O–H absorption band at 3350 cm^−1^. It can be assumed that the OH group is engaged in the formation of hydrogen bonds or complexation with metal nanoparticles.

**Figure 4:**
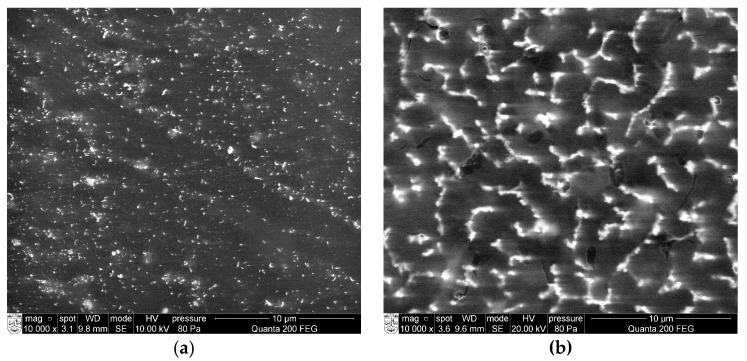
SEM micrographs of silver-containing UV-cured aliphatic silicone acrylate coating: (**a**) 0.5 wt % silver nanoparticles; (**b**) 1.43 wt % silver nanoparticles.

**Figure 5:**
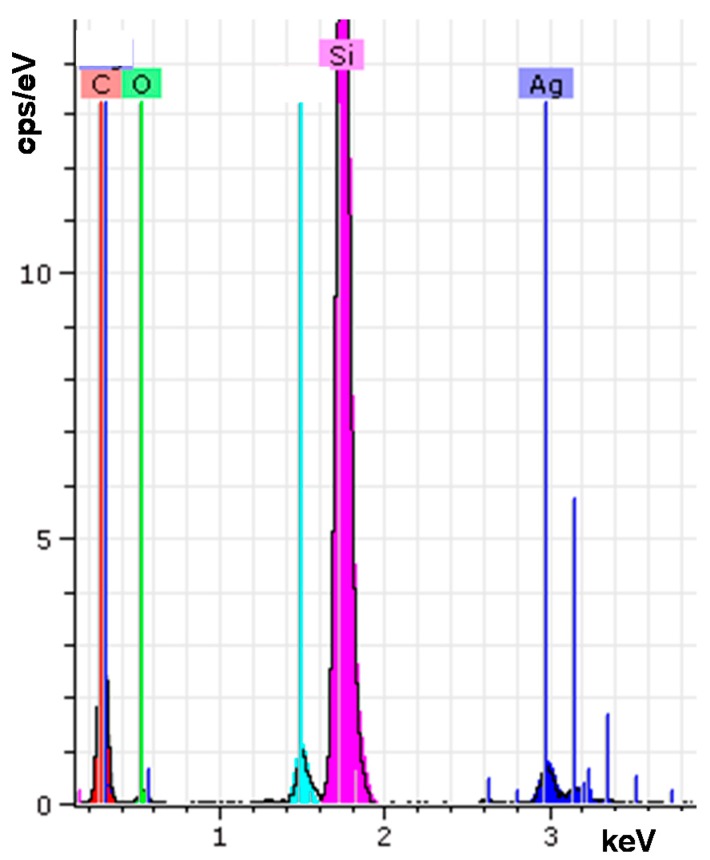
EDS spectrum of silver containing (1 wt %) UV-cured aliphatic silicone acrylate coating.

The surface morphologies of the silicone acrylate–silver nanocomposites were investigated by scanning electron microscopy (SEM) and energy dispersive X–ray spectroscopy (EDS) analysis. The presented SEM micrographs (Figure 4) clearly show the formation of silver nanoparticles in the aliphatic silicone acrylate network. Silver nanoparticles are well dispersed in the polymer matrix. However, the increase of silver nanoparticles concentration causes the accumulation of nanoparticles, which facilities the formation of clusters (Figure 4b). The EDS spectrum of the silicone acrylate and silver nanocomposite in Figure 5 also confirmed the formation of silver nanoparticles in the silicone acrylate matrix.

**Figure 6:**
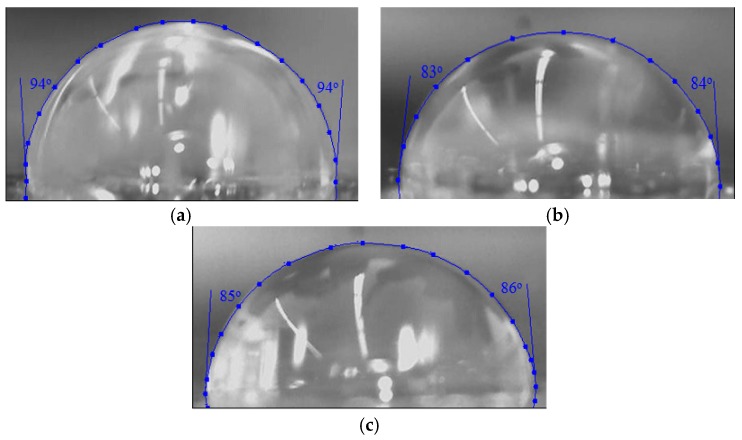
Five microliter water droplet on UV-cured aliphatic silicone acrylate coating (**a**) without nanoparticles and containing (**b**) 0.5 wt % and (**c**) 1.43 wt % of silver nanoparticles.

Changes of surface free energy due to the incorporation of silver nanoparticles into the aliphatic silicone acrylate coating were determined using contact angle (CA) measurements. The 5 μL water droplet measurements were employed (Figure 6). The UV-cured aliphatic silicone acrylate was found to be hydrophobic (CA = 94° ± 1°). The formation of silver nanoparticles in aliphatic silicone acrylate during the UV-curing process reduces the contact angle, because metallic silver particles have a large surface energy. Importantly, CA values do not depend on the nanoparticles concentration—an increase of the content of silver nanoparticles from 0.5 to 1.43 wt % resulted in a CA value of 84 ± 2°. It is possible that with the increase of metallic silver concentration the nanoparticles tend to accumulate, thereby creating clusters. The smaller the silver particles, the higher are their surface energy and surface activity. According to [40], polymer composites exhibit higher contact angle when the concentration of silver nanoparticles is increased due to the formation of silver clusters of large dimensions.

### 2.2. Antibacterial Activity

**Figure 7:**
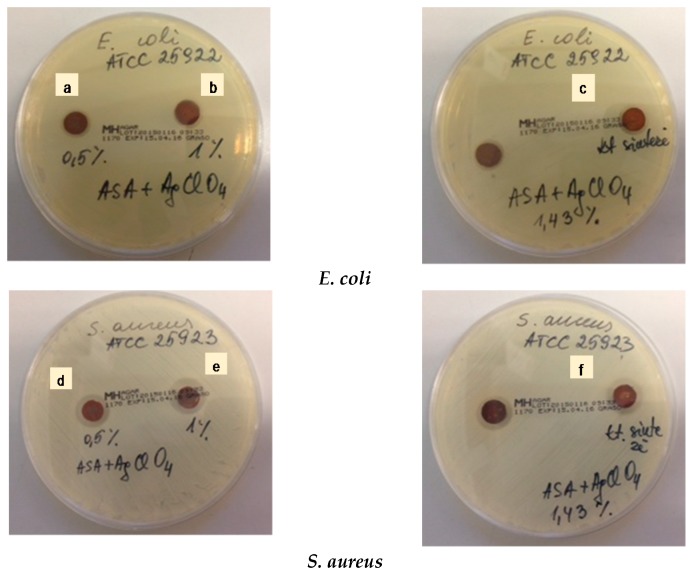
Antibacterial activity against *E. coli* and *S. aureus* of UV-cured silicone acrylate composite samples with different concentration of silver nanoparticles: (**a**,**d**) 0.5 wt %; (**b**,**e**) 1 wt %; (**c**,**f**) 1.43 wt %.

The antibacterial activity of the hybrid silver nanoparticles containing UV-cured aliphatic silicone acrylate coatings obtained via in situ formation method was investigated by different methods. At first, qualitative assessment of the antibacterial activity was used by measuring the diameter of the growth inhibition zone. Disks of hybrid silicone acrylate films were placed on the agar plates inoculated with the Gram-negative bacteria *Escherichia coli* ATCC 25922 and the Gram-positive bacteria *Staphylococcus aureus* ATCC 25923*.* Figure 7 illustrates the antibacterial activity of the silicone acrylate organic–inorganic hybrid coatings containing different amounts of silver nanoparticles (from 0.5 to 1.43 wt %). The antibacterial effect increased gradually as the silver nanoparticles concentration increases. The zone of inhibition for *E. coli* increased by 20–30%, while for *S. aureus* this increase was noticeably higher, at 60–90% (Table 1). Importantly, silver nanoparticles synthesized during silicone acrylate polymerization process acted as an effective antibacterial agent.

**Figure 8:**
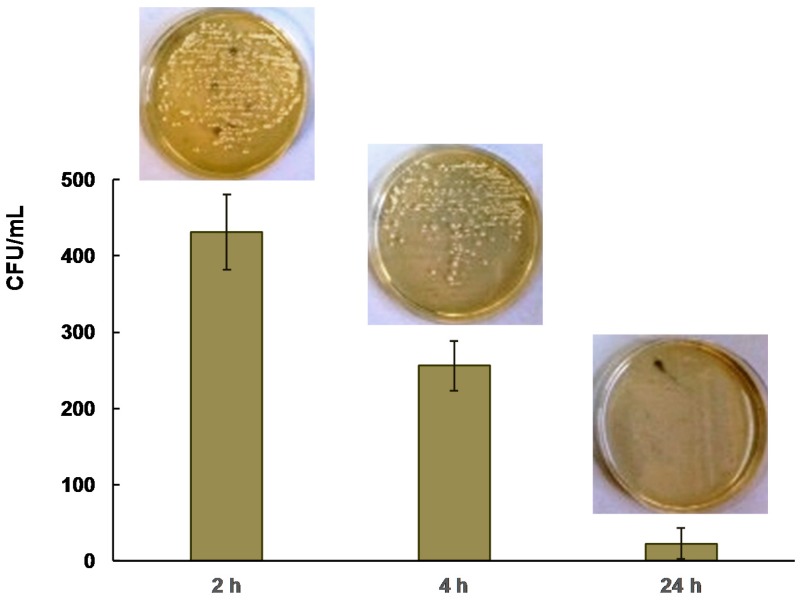
Bactericidal effect of UV-cured silicone acrylate coating containing 1.43% of silver nanoparticles on *S. aureus* after different contact time.

The growth inhibition around the hybrid coating disks created not large inhibition zones. This may be due to the incomplete physical contact between the silver nanoparticles in the hybrid coating and the surrounding culture medium. Therefore, in the second round, a direct contact test was performed for quantification of the bactericidal effect. A count of the colonies revealed that the UV-cured aliphatic silicone acrylate organic–inorganic composite coating significantly inhibited the growth of *S. aureus*, as shown in Figure 8. The bacteria decreased to zero in three out of four repetitions after incubation for 24 h.

The antibacterial effect could be the result of the dissociation of silver nanoparticles into Ag^+^ ions and their accumulation on the coating surface. Silver ions accumulate on the bacterial cell surface, which interacts with the microbial membrane to cause structural change, permeability, and finally bacterial cell death [31]. The influence on bacteria viability depends extremely on the size, shape, and concentration of nanoparticles [32,33]. In [34], it is reported that silver nanoparticles accumulation on the *E. coli* cell membrane makes gaps in the entirety of the bilayer, which predisposes it to the increased penetrability and finally bacterial cell death [31].

**Figure 9:**
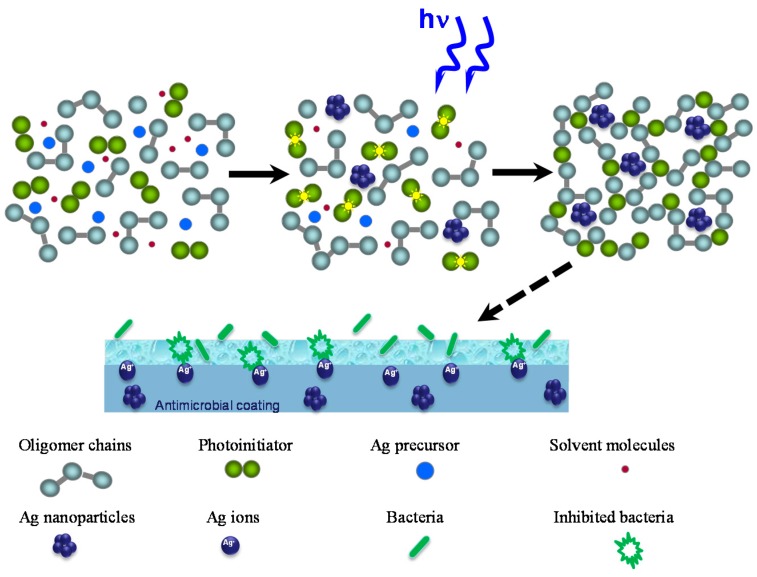
Scheme of the formation of silver nanoparticles containing UV-cured aliphatic silicone acrylate coating and possible bacterial inactivation mechanism via interaction with antibacterial coating surface.

The model of the silicone acrylate formation with simultaneous conversion of silver perchlorate to silver nanoparticles and possible bacterial inactivation mechanism is presented in Figure 9.

According to the studies of other researchers, silver nanoparticles possess a strong antibacterial and antiviral activity. Acting with microorganisms, they impact the growth of bacterial biofilms. Silver nanoparticles interact with bacterial surfaces, as well as with their particular structure [22,23,24,25,26,27,28,29]. When the size of silver nanoparticles is larger than 10 nm, the predominant bacteria inactivation mechanism is through silver ions [30]. Although nanoparticles’ antibacterial effects have been described in detail, their mechanism of action still requires further elucidation both from chemical and biological points of view.

## 3. Materials and Methods

### 3.1. Materials

Bifunctional aliphatic silicone acrylate oligomer with viscosity of 50–70 Pas, suitable for use in UV and electron beam curing composites (CN9800), was purchased from Sartomer (Arkema Group, Colombes Cedex, France). Its polymerization was carried out using mixture bis(2,6-dimethoxybenzoyl)-2,4,4-trimethyl pentylphosphineoxide and 2-hydroxy-2-methyl-1-phenyl-propan-1-one in a ratio of 1:3 (Irgacure 1700) as photoinitiator, supplied by BASF (Southfield, MI, USA). Silver perchlorate AgClO_4_ (Ag > 50.5%) and acrylic acid were purchased from Sigma Aldrich (St. Louis, MO, USA).

### 3.2. Nanocomposite Preparation

**Figure 10:**
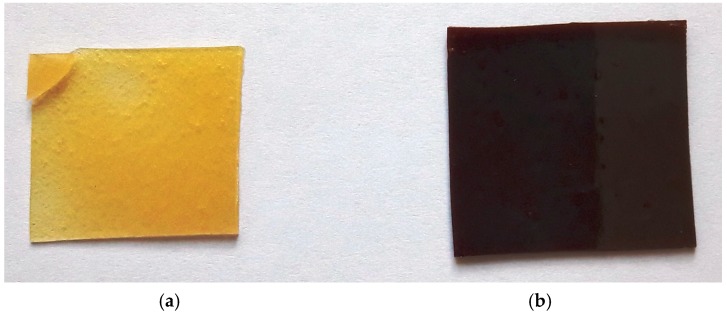
Aliphatic silicone acrylate coating films: (*a*) without silver nanoparticles; (*b*) with silver nanoparticles.

Silver perchlorate salt (0.05–0.15 g) was first dissolved in 1.00 mL of acrylic acid, and then 0.1–0.3 g of photoinitiator Irgacure 17,000 was added (in ratio AgClO_4_:photoinitiator = 1:2). The mixture was continuously stirred at ambient temperature until a homogeneous solution was obtained. Silver nanoparticles precursor, solvent, and photoinitiator mixture were mixed with 4.00 g of bifunctional aliphatic silicone acrylate oligomer CN9800 for 10 min at ambient temperature until a homogeneous suspension was formed and then kept under vacuum for 10 min at ambient temperature to remove air bubbles. After that, the obtained mixture was poured onto a glass plate. The polymerization and silver salt photoreduction to silver nanoparticles initiated by Irgacure 1700 was carried out with a medium pressure mercury lamp (1 kW, Hibridas Photosensitive Paste UV Exposure Unit MA-4). After irradiation of the composition for 120 s, silicone acrylate coatings having a thickness of 1 mm without or with silver nanoparticles of 0.5–1.43 wt % concentration were formed. Higher silver nanoparticles concentration in polymer matrix via in situ photo-reduction method becomes problematic. Visual observation of the resultant materials shows that the yellowish films without silver nanoparticles have a good optical transparency; meanwhile, those with embedded silver nanoparticles furnish a brown coloration (Figure 10).

### 3.3. Nanocomposite Characterization

An optical spectrometer Avantes that is composed of a deuterium halogen light source (AvaLight DHc, Avantes, Apeldoorn, The Netherlands) and spectrometer (Avaspec-2048, Avantes, Apeldoorn, The Netherlands) was used to record UV-visible light absorbance spectra. The analysis was performed in the wavelength range of 337–840 nm.

The structural peculiarities of the synthesized silver nanoparticles was determined using X-ray diffractometer D8 Discover (Bruker AXS GmbH, Billerica, MA, USA) with parallel beam focusing geometry of Cu Kα_1_ (λ = 0.154 nm) radiation. Processing of the resultant diffractograms was performed with DIFFRAC.EVA software (version 3.0, Billerica, MA, USA). Phase identification and interpretation involved matching the diffraction pattern of free-standing silver nanoparticles containing silicone acrylate coating to patterns of single-phase reference materials. Reference patterns from the Joint Committee on Powder Diffraction Standards–International Centre for Diffraction Data (JCPDS–ICDD) database were used.

The Fourier transform infrared (FTIR) spectra were obtained by using Spectrum GX FTIR spectrometer (Perkin-Elmer, Waltham, MA, USA) equipped with a horizontal attenuated total reflection (HATR) accessory. The HATR FTIR spectra of samples were recorded at room temperature in the wavenumber range of 4000–650 cm^−1^ with a resolution of 1 cm^−1^. Each spectrum was averaged from 16 scans at a scan rate of 0.2 cm∙s^−1^. Collected spectra were processed with the Spectrum^®^ v5.0.1 software from Perkin-Elmer (Waltham, MA, USA).

The CA measurements were performed at room temperature (23 °C) using sessile drop method. One droplet of deionized water (~5 μL) was deposited on the sample surface. Optical images of the droplet were obtained, and contact angle was measured using a method based on B-spline snakes (active contours).

SEM analysis of coatings structure was performed with a microscope Quanta 200 FEG (FEI, Eindhoven, The Netherlands) operating in a low vacuum at 20.0 kV using an LDF detector. The chemical analysis of nanocomposites was performed by EDS technique with a Bruker XFlash 4030 detector (Berlin, Germany) (accelerating voltage 10 kV, distance between the bottom of the objective lens and the object 10 mm).

### 3.4. Antibacterial Studies

The antibacterial activities of the silver nanoparticles containing UV-cured aliphatic silicone acrylate organic–inorganic composite coatings were tested using standard strains of *E. coli* and *S. aureus*. Two methods—the agar disk diffusion test and the direct contact test—were chosen for evaluation of antibacterial activity.

For the disk diffusion procedure, *E. coli* and *S. aureus* were cultured in Tryptone Soya Broth (TSB) solution. Following that, about 1 mL of the bacteria was pipetted from the overnight phase into another flask with 30 mL of freshly-prepared TSB. Bacteria were grown at 37 °C in the incubator shaker for another 5 h to obtain bacteria with higher activity and more viability than in other growth phases. Commercially-available Mueller-Hinton agar plates were used in antibacterial testing. The stationary phase bacterium was diluted into a concentration of about 10^3^ CFU/mL with NaCl solution (0.85%). Diluted suspensions (0.1 mL) of the previously mentioned microorganisms were transferred and spread onto the solid surface of the plate. In the inhibition experiment, the silver nanoparticles containing silicone acrylate discs were placed on Mueller-Hinton agar plates inoculated with *E. coli* and *S. aureus* and incubated at 37 °C for 24 h. Later, the zone of inhibition was noted and tabulated. Three replicates were carried out for each testing.

To quantify the antimicrobial activity of the hybrid composite, a modified direct contact test was used [41]. Overnight cultures were diluted into a concentration at 10^5^ CFU/mL with 0.85% NaCl suspension. The bacterial suspension (0.1 mL) was deposited on glass slides and covered with thin film (thickness ca. 0.4 mm; dimension 25 mm × 25 mm) of UV-cured silicone acrylate to facilitate direct contact of the bacteria with the silver nanoparticles on the acrylate silicone composite surface. Two-layer assembly with the liquid film containing bacteria were placed in petri plate containing 1 mL of phosphate-buffered saline (PBS) to maintain a damp environment and incubated at room temperature for 3, 6, and 24 h. After that, 9 mL of PBS was added into each petri plate to detach the assembly of glass slide and polymer film by soft shaking. Lastly, the bacterial suspensions were grown in nutrient agar medium to count colonies and calculate CFU/mL. Lower than 500 bacterial counts in the culture medium were attributed to the antibacterial activity of the investigated hybrid coating. Four repetitions were carried out for each testing.

## 4. Conclusions

The aliphatic silicone acrylate composites containing silver nanoparticles can be easily synthesized via in-situ photopolymerization technique. The formation of a polymeric matrix and the conversion of silver salt into silver nanoparticles proceeds simultaneously. Silicone acrylate composites with 0.5–1.43 wt % of silver nanoparticles were obtained. Silicone acrylate polymer OH group is engaged in the formation of hydrogen bonds or complexation with silver nanoparticles. The synthesized silver nanoparticles having a size of 20 ± 5 nm are homogeneously dispersed in silicone acrylate hybrid coating. Direct photoreduction of silver salt by UV-radiation in the organic media produced silver nanoparticles exhibiting cubic crystal structure with space group Fm-3m. Silver nanoparticles increased the surface energy of the silicone acrylate hybrid coating. Aliphatic silicone acrylate hybrid coatings exhibited antibacterial activity, and can be used to protect against bacterial invasion. The predominant bacteria inhibition mechanism is probably through silver ions, as the size of the synthesized silver nanoparticles is larger than 10 nm.

## Figures and Tables

**Table:** 

Element	wt %	at %	Error in %
Carbon K	41.53	61.16	6.79
Oxygen K	10.81	11.95	3.47
Silver L	6.87	1.13	0.19
Silicon K	38.48	24.24	1.63

**Table 1:** Mean inhibition zone of silver nanoparticles containing UV-cured silicone acrylate coating against different pathogens presented as an interval range.

Silver Nanoparticles Concentration (wt %)	Inhibition Zone *	
	*E. coli*	*S. aureus*
0	0	0
0.5	1.14–1.27	1.61–1.66
1.0	1.15–1.34	1.82–1.94
1.43	1.26–1.34	1.83–2.01

Notes: * Three replicates were carried out for each testing.
